# Effects of Maternal Exposure to Ultrafine Carbon Black on Brain Perivascular Macrophages and Surrounding Astrocytes in Offspring Mice

**DOI:** 10.1371/journal.pone.0094336

**Published:** 2014-04-10

**Authors:** Atsuto Onoda, Masakazu Umezawa, Ken Takeda, Tomomi Ihara, Masao Sugamata

**Affiliations:** 1 Department of Hygienic Chemistry, Faculty of Pharmaceutical Sciences, Tokyo University of Science, Noda, Chiba, Japan; 2 Department of Pathology, Tochigi Institute of Clinical Pathology, Nogi, Tochigi, Japan; 3 The Center for Environmental Health Science for the Next Generation, Research Institute for Science and Technology, Tokyo University of Science, Noda, Chiba, Japan; Virginia Commonwealth University, United States of America

## Abstract

Perivascular macrophages (PVMs) constitute a subpopulation of resident macrophages in the central nervous system (CNS). They are located at the blood-brain barrier and can contribute to maintenance of brain functions in both health and disease conditions. PVMs have been shown to respond to particle substances administered during the prenatal period, which may alter their phenotype over a long period. We aimed to investigate the effects of maternal exposure to ultrafine carbon black (UfCB) on PVMs and astrocytes close to the blood vessels in offspring mice. Pregnant mice were exposed to UfCB suspension by intranasal instillation on gestational days 5 and 9. Brains were collected from their offspring at 6 and 12 weeks after birth. PVM and astrocyte phenotypes were examined by Periodic Acid Schiff (PAS) staining, transmission electron microscopy and PAS-glial fibrillary acidic protein (GFAP) double staining. PVM granules were found to be enlarged and the number of PAS-positive PVMs was decreased in UfCB-exposed offspring. These results suggested that in offspring, “normal” PVMs decreased in a wide area of the CNS through maternal UfCB exposure. The increase in astrocytic GFAP expression level was closely related to the enlargement of granules in the attached PVMs in offspring. Honeycomb-like structures in some PVM granules and swelling of astrocytic end-foot were observed under electron microscopy in the UfCB group. The phenotypic changes in PVMs and astrocytes indicate that maternal UfCB exposure may result in changes to brain blood vessels and be associated with increased risk of dysfunction and disorder in the offspring brain.

## Introduction

Resident phagocytes of the central nervous system (CNS) are categorized into four types: microglia, meningeal macrophages, choroid plexus macrophages, and perivascular macrophages (PVMs). These contribute to maintaining homeostasis in the CNS [1], [2]. Recently initiated investigations aim to elucidate the characteristics and function of PVMs, also called perivascular cells, perivascular microglia and fluorescent granular perithelial cells [3]. PVMs are localized in the perivascular space (Virchow-Robin space) and are surrounded by the vascular endothelial basement membrane and glia limitans [4], [5]. In this space, PVMs adjoin endothelial cells, pericytes [4], [5], [6], and the end-foot of astrocytes, which are one of the types of cerebral parenchyma glial cells in the surrounding brain microvessels [7], [8]. This space plays a particularly important role in the drainage of interstitial fluid containing unnecessary substances and waste including β-amyloid from the central grey matter and cerebral cortex [9], [10], [11], [12]. PVMs encounter various substances, including pathogens and waste from blood flow and brain parenchyma, and play a crucial role in regulating inflammatory responses in the CNS [13]. PVMs are the only cells that display constitutive phagocytic potential in the brain parenchyma [14] that and express immunophenotypical markers of activation such as major histocompatibility class II (MHC II), B7, CD40, and Fc receptor (FcR) [3]. Because of their unique localization, phagocytic function and character PVMs are also essential for maintaining blood brain barrier (BBB) function [15].

Research focused on PVM turnover reported that PVMs are partially and continuously replaced under physiological conditions [3]. Under pathological conditions such as rodent experimental allergic encephalomyelitis, there is a great increase in the number of PVMs accumulated around blood vessels in the brain [8]. Previous studies have also suggested that PVMs respond uniquely to particulate matter. In a rat model, carbon particles injected into the cerebral ventricle were move to perivascular spaces and phagocytosed by PVMs within 1 week. Moreover, the carbon could be detected for more than two years in cells that were laden with particles [14]. These data indicated that it is difficult for PVMs to excrete or dispose of carbon particles and that the presence of these particles may induce some signals or responses in the surrounding cells. This evidence suggested that in PVMs and their surrounding perivascular spaces, particulate substances may stay and promote biological responses over a long period of time.

The authors aimed to investigate the effects of nano-sized particles because previous studies suggested that they have a greater effect than larger sized particles [16], [17], [18], especially in the CNS [19] and endothelial cells [20], [21]. Because of their small size, nano-sized particles have a larger relative surface area per mass than do bulk-size particles of the same material; this feature often makes nano-sized particles more toxic [22]. Small size of nano-sized particles also enables certain nano-sized particles to cross cell membranes and translocate from the environment into the organism [23]. The lungs and airways are the most important exposure sites for involuntary exposure to nano-sized particles. Respirable nano-sized particles not only elicit local pulmonary effects [24], [25], [26], but they also can translocate from lung epithelium to extrapulmonary organs [27], [28] and developing fetus [29]. It has also been reported that maternal exposure to diesel exhaust particles, which contain nano-sized carbon particles at their core, alters the ultrastructure of PVMs and surrounding tissues in the brain of mouse offspring [30]. This finding suggests that maternal exposure to nano-sized particles may alter the phenotype of PVMs and other cerebral cells in mouse offspring.

The aim of the present study was to investigate the effects of maternal exposure to ultrafine carbon black (UfCB) on the perivascular regions, especially the PVMs and astrocytes close to blood vessels in the brains of the offspring of maternally exposed mice (UfCB-exposed offspring).

## Materials and Methods

### Ultrafine carbon black

Printex 90, purchased from Degussa Ltd. (Frankfurt, Germany), was used as UfCB. The particle is insoluble in water [31]. The manufacturer reported an average primary particle size of 14 nm and an organic impurity content of less than 1%. The specific surface area was determined to be 295-338 m^2^/g [32]. The total carbon content measured was >99 wt%, with 0.82 nitrogen and 0.01 hydrogen wt%. Very low levels of both total polycyclic aromatic hydrocarbon (PAH) (74.2 ng/g) [33], [34] and total endotoxin (0.142 EU/mg Printex 90) were detected in the sample.

The UfCB particles were suspended at 5 mg/mL in distilled water, sonicated for 30 min, and then filtered through a 450-nm filter (S-2504; Kurabo Co. Ltd., Osaka, Japan) immediately before administration. The particles in the filtered suspension were characterized by transmission electron microscopy (TEM; JEM 1200EXII, JEOL Ltd., Akishima, Tokyo, Japan) on collodion-coated 200 Cu mesh (Nisshin EM, Cat.No. 6511). The size distribution of secondary UfCB particles in the suspension was determined by dynamic light scattering measurement using a NANO-ZS (Sysmex Co., Kobe, Hyogo, Japan) and the Rayleigh-Debye equation. The UfCB concentration in the suspension was calculated to be 95 μg/mL by peak area of carbon signal (2.77 keV) obtained using a field emission scanning electron microscope (JSM-6500F) with an attached energy-dispersive X-ray analyzer (JSM-6500F).

### Animals and treatments

**Figure 1:**
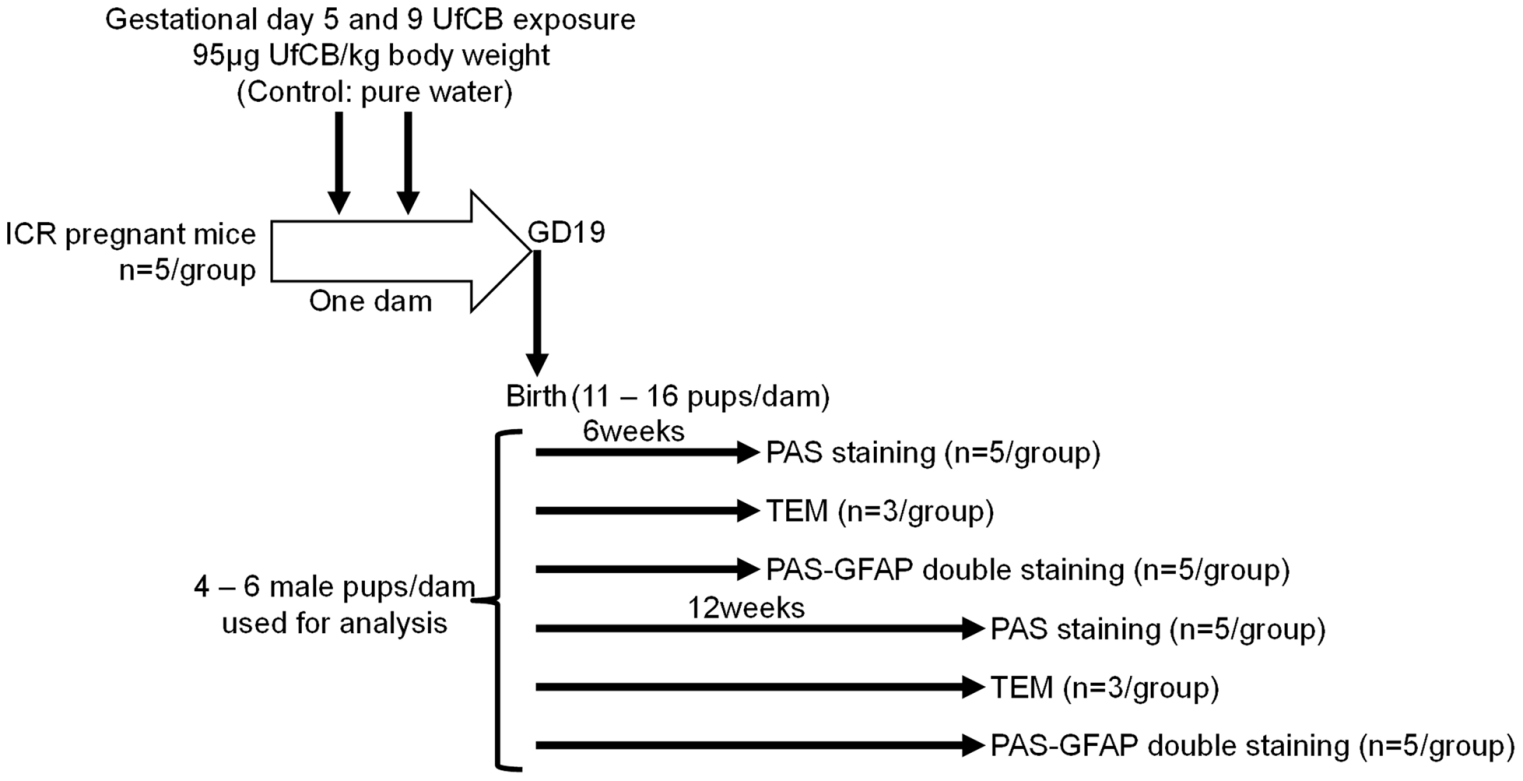
Summarised scheme of animal treatments and sample collection.

Ten pregnant ICR mice (11 weeks of age) were purchased from SLC Inc. (Hamamatsu, Shizuoka, Japan) and were randomly divided into two groups: UfCB-exposed (n = 5) and control (n = 5). The mice were housed under controlled temperature (23±1°C) and humidity (55%±5%) with a 12-hr dark/light cycle and ad libitum access to food and water. The pregnant mice were put into anesthesia box filled with halothane, and then taken out from the box when they began to sleep. The mice were immediately laid on their back and treated with 1 mL/kg (body weight) of UfCB suspension (95 μg/mL, for UfCB group) or distilled water (for control group) by intranasal instillation into both nostrils. 95 μg/mL was maximum concentration of UfCB suspension without bulk agglomeration or any dispersant. The total dose of UfCB (190 μg/kg body weight) was lower than the doses used in many earlier studies of nano-sized particle effects. The treatment were perfomed on gestational days (GDs) 5 and 9, because the fetus of mice on these days is particularly sensitive to various foreign substances in comparison to any other fetal period [35]. Previous study showed that fetal malformation were observed by maternal exposure to single-wall carbon nanotube on GD 5.5 [36]. Additionally, developmental effect of respiratory exposure to UfCB on kidney was examined by twice intranasal instillation on GDs 5 and 9 in the previous study [37]. The number of pups per dam was adjusted randomly to 11 or 12 on postnatal day 1. After weaning at 3 weeks of age, 4–6 male offspring per dam were randomly selected and used for analysis. Brains were collected from male offspring mice at 6 and 12 weeks after birth (Figure 1). All experiments were performed in accordance with Animal Research: Reporting In Vivo Experiments guidelines for the care and use of laboratory animals [38] and were approved by Tokyo University of Science's Institutional Animal Care and Use Committee. All sampling was performed under sodium pentobarbital (50 mg/kg) anesthesia, and all efforts were made to minimize suffering.

### Periodic Acid Schiff (PAS) staining

Brains from 6-week-old (n = 5/group) and 12-week-old (n = 5/group) male offspring mice were fixed in 4% paraformaldehyde and 1% glutaraldehyde, embedded in paraffin and cut into 3-μm sections for analysis with PAS staining to visualize PVM granules. The sections were oxidized in 1% periodic acid solution for 1 min. After rinsing for 3 min in distilled water, the sections were soaked in cold Schiff reagent for 45 min. Next, the sections were soaked in sulphurous acid solution 3 times for 5 min and then rinsed for 3 min in distilled water. Finally, the sections were counterstained in haematoxylin for 1 sec, then washed in flowing tap-water, dehydrated in graded alcohol, cleared in xylene, and coverslips were applied with permount mounting medium (Thermo Fisher Scientific Inc., Weltham, MA,USA).

### PAS-positive PVM counting

PAS-positive PVMs were observed in sections using a BX-10 microscope (Olympus, Co., Tokyo, Japan) equipped with a digital camera (BX41; Olympus). Fifty sections (total 150 μm) from the longitudinal fissure of the cerebrum along sagittal plane were prepared from each mouse. One in every 10 sections was chosen (every 30 μm) for analysis by PAS staining. In total, 3 sections per mouse (about 150 μm^2^/mouse) were subjected to quantitative analysis (Table S1). Stained sections were photographed by optical microscope at 40X magnification. PAS-positive PVM was confirmed by optical microscope with 400X magnification and plotted on 40X-magnified photographs. The PAS-positive cell number per 1 mm^2^ area was calculated for each region.

### Transmission electron microscopy (TEM)

Brains which removed from 6 (n = 3/group) and 12 (n = 3/group) week-old male offspring mice were fixed by 4% paraformaldehyde and 1% glutaraldehyde. Fixed tissues were washed with phosphate buffer (pH7.4) and post-fixed with osmium tetroxide. These tissue samples were dehydrated in a graded series of ethanol and propylene oxide, and then embedded in epoxy resin (Epon 812, Shell Chemicals Ltd., Houston, TX, USA). Ultrathin sections of each sample were stained with uranyl acetate and lead citrate and examined by transmission electron microscopy (JEOL100-S, JEOL Ltd., Tokyo, Japan) with an accelerating voltage of 80 kV.

### Double-staining for GFAP and PAS-positive granules

Brains from 6-week-old (n = 5/group) male offspring mice were used for double-staining for glial fibrillary acidic protein (GFAP) and PAS-positive granules. The brain samples were fixed for 24 hr in 4% paraformaldehyde. Right brains were embedded in paraffin and cut into 6-μm sections for wide-field analysis. Left brains were cryoprotected in phosphate-buffered sucrose solutions (10% sucrose, 4–6 hr; 20% sucrose, 4–6 hr; 30% sucrose, 12–36 hr) with 0.1% sodium azide, embedded in Tissue-Tek OCT compound (Sakura Finetek Japan Co., Ltd., Tokyo, Japan) and then cut into 10-μm frozen sections for observation of detailed morphology.

Visualization of GFAP and PAS-positive granules was performed on paraffin and frozen sections using antibodies and an avidin–biotin-peroxidase method. After blocking endogenous peroxidase and preincubation in 10% normal horse serum, sections were incubated in primary rabbit polyclonal anti-GFAP antibody (Code-No. Z0334, DakoCytomation, Copenhagen, Denmark) diluted 1∶1000 in 0.1 M PBS with 0.1% Trion X (PBS-Tx) for 16 hr at 4°C. After rinsing 3 times for 5 min in PBS-Tx, sections were further incubated in secondary biotinylated donkey anti-rabbit IgG (AP182B, Chemicon, Temecula, CA, USA; 1∶1000) for 120 min at room temperature and rinsed 3 times for 5 min in PBS-Tx. The sections were oxidized in 1% periodic acid solution for 3 min, rinsed for 1 min in distilled water, and then soaked in cold Schiff reagent for 60 min. Next, the sections were soaked in sulphurous acid solution 3 times for 3 min and then rinsed for 1 min in distilled water. Finally, the sections were treated with an avidin-biotin-peroxidase complex (Vectastain ABC peroxidase kit, Vector Laboratories Inc., Burlingame, CA, USA; 1∶400) for 120 min. The sections were then reacted for peroxidase activity in a solution of 0.02% 3,3′-diaminobenzidene (DAB) in 0.1 M Tris–HCl buffer (pH 7.6) and 0.01% H_2_O_2_ for 20 min. Immunoreactivity for GFAP was localized to the astrocytic cytoplasm and was visible as light-brown staining. Sections were then washed in 0.1 M PBS, dehydrated in graded alcohol, cleared in xylene, and coverslips were applied with permount mounting medium (Thermo Fisher Scientific).

### Statistical analysis

Data are expressed as the mean ± SD. The effects of maternal exposure to UfCB on number and sex ratio of pups at birth were identified by an unpaired t-test, and their body weight at 6 and 12 weeks of age were identified by 2-way ANOVA (Exposure × Age) followed by the Tukey-Kramer post hoc test. For quantitative observation of PAS-positive PVMs, an unpaired t-test was performed. Data analysis was performed using Excel Statistics 2012 for Windows (Social Survey Research Information, Tokyo, Japan). Significance of the difference among means was estimated at P<0.05.

## Results

### UfCB characterisation

**Figure 2:**
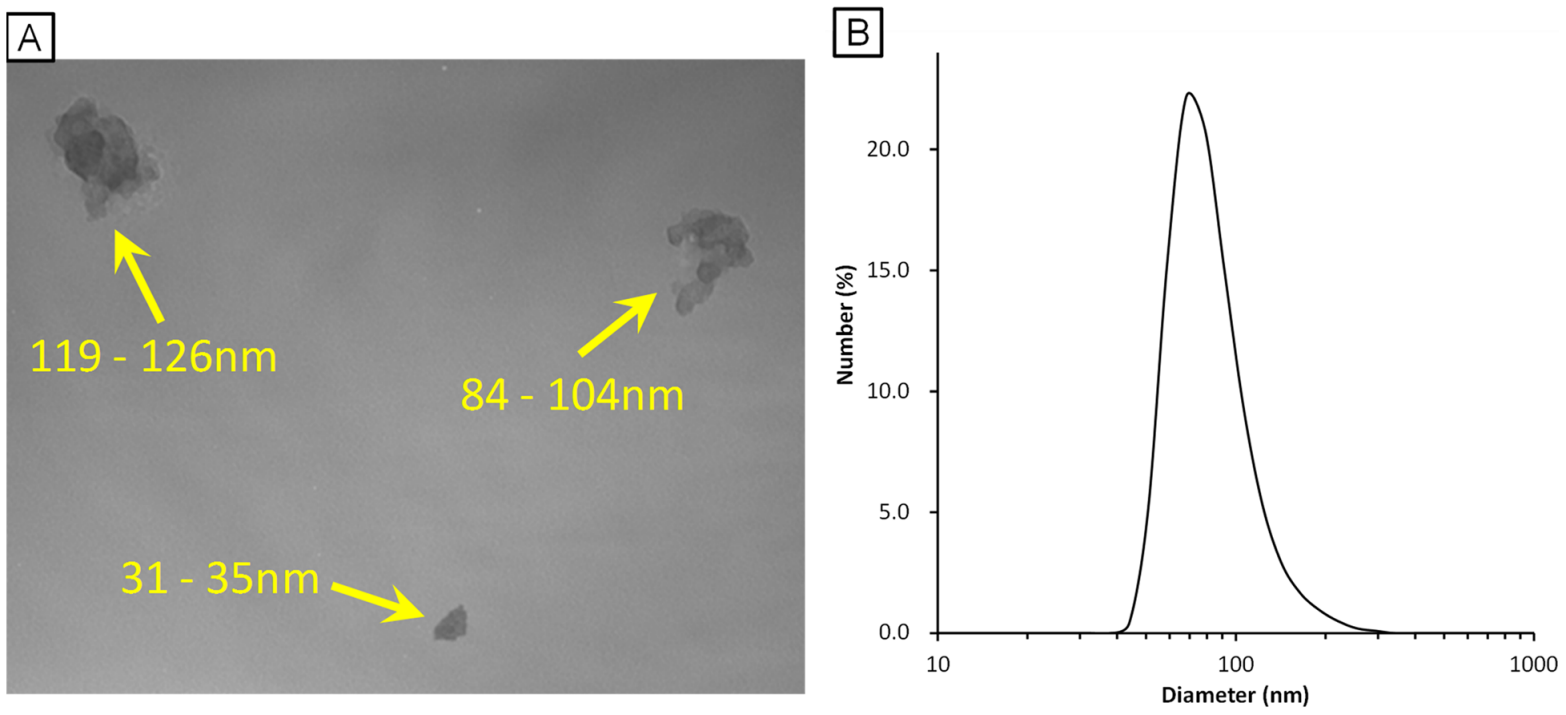
Characterisation of ultrafine carbon black (UfCB). A: Transmission electron microscopy images of UfCB particles in the instillation medium. The yellow numerical value means minor axis - major axis of the secondary UfCB particles. B: Dynamic light scattering data of UfCB in the instillation medium.

The particle suspension (95 μg/mL) was characterised by transmission electron microscopy (TEM) and dynamic light scattering analysis. The TEM analysis of the instillation suspension showed that Printex 90 consisted of open chain-agglomerates of 30–200 nm in diameter (Figure 2A). The filtered Printex 90 suspension, which was employed for intranasal instillation, showed the presence of small agglomerated particles with a peak size of 84.2 nm (Figure 2B). The polydispersity index of 0.143 was low, indicating a narrow size-distribution. This 84.2 nm size corresponds well with the typical small agglomerate sizes of Printex 90 observed under TEM (Figure 2A).

### Number and body weight of offspring

There was no significant difference between control and UfCB-exposed offspring in number and sex ratio of pups at birth (Table 1) or their body weight at 6 and 12 weeks of age (Table 2).

**Table 1:** Number and sex ratio of offspring.

	Number of dams	Number of offspring	Sex ratio (%)*
**Control**	5	13.8±2.2	46.8±9.8
**UfCB**	5	13.0±1.2	62.5±21.4

Data are presented as mean ± SD.

*Sex ratio (%)  =  male/(male + female) ×100

**Table 2:** Effect of maternal exposure to UfCB on body weight (g) of male offspring at 6 and 12 weeks of age.

	6-week-old male	12-week-old male
**Control**	33.9±3.0	42.8±3.3
**UfCB**	33.9±2.1	40.8±5.2

Data are presented as mean ± SD

### Light-microscope examination of PAS staining

**Figure 3:**
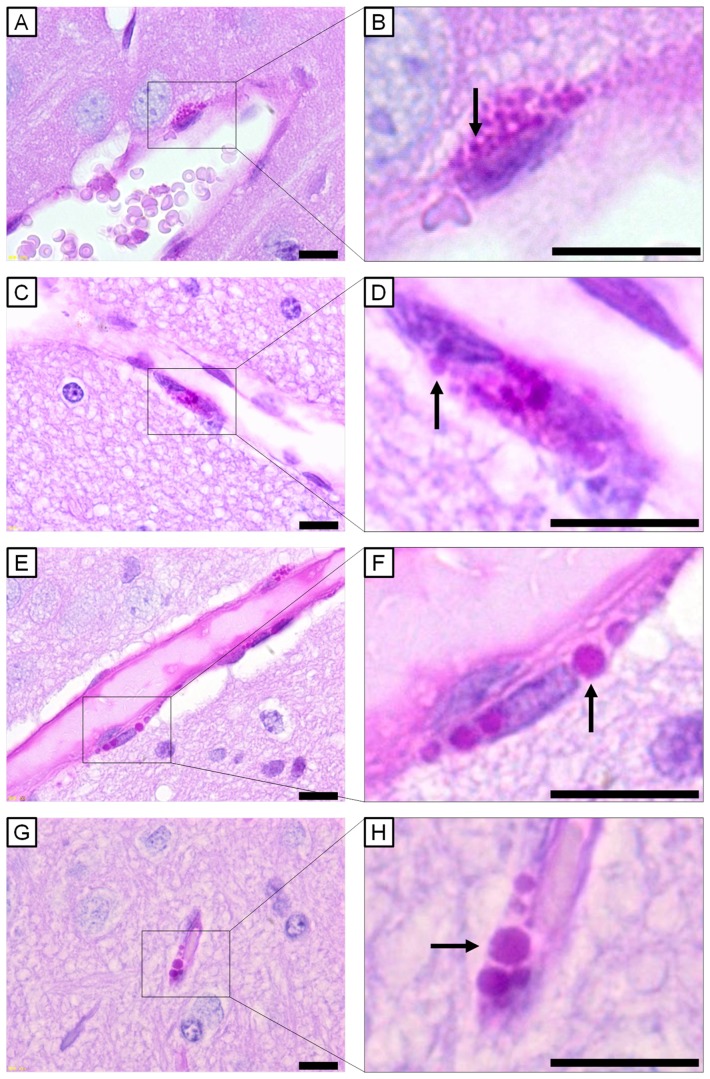
Light micrographs of perivascular macrophages stained with PAS-haematoxylin. All scale bars represent 10 μm. Perivascular macrophages (PVMs) surrounding cerebral blood vessels of (A, B) 6-week-old control mouse, (C, D) 12-week-old control mouse, (E, F) 6-week-old UfCB-exposed offspring mouse, and (G, H) 12-week-old UfCB-exposed offspring mouse were shown. B, D, F, H: Enlarged view of A, C, E and G: PVMs of control mice contained many PAS-positive granules, sized 0.9 μm (B, arrow) and 1.3 μm (D, arrow), in the cytoplasm. Many PAS-positive granules were enlarged in UfCB-exposed offspring (F, arrow: 2.6 μm; H, arrow: 3.0 μm).

The intracellular granule, a feature of PVMs, was stained red with PAS [39]. For comparing the general characteristics and contents of PVMs in the offspring of maternally UfCB-exposed mice to control mice, 20 paraffin sections per mouse were observed under a light microscope. The colour photograph shows representative findings for PVMs in both control mice and UfCB-exposed offspring (Figure 3).

In control mice, PVMs were seen exclusively around vessels and located abluminal to endothelial cells in both 6- and 12-week-old offspring (Figure 3A, C). PVMs surrounding cerebral microvessels were slender in shape and contained many PAS-positive granules in their cytoplasm. The diameter of most of the PAS-positive granules was about 1 μm (Figure 3B, D). PAS-positive intracellular inclusions were stained weakly, and the contours of PAS-positive granules were occasionally obscure (Figure 3A, C).

In UfCB-exposed mice, the PVMs were similar in size and shape to those of the control mice, but the diameter of PAS-positive granules was larger (2–3 μm) (Figure 3F, H). Furthermore, the number of PAS-positive granules per PVM was decreased in UfCB-exposed offspring at both 6 weeks and 12 weeks of age (Figure 3F, H).

### Quantitative observation of PAS-positive PVMs

For comparing the number of PVMs in UfCB-exposed offspring to control, a total of 15 paraffin sections per group were stained with PAS-haematoxylin and PAS-positive PVMs were counted under a light microscope.

**Figure 4:**
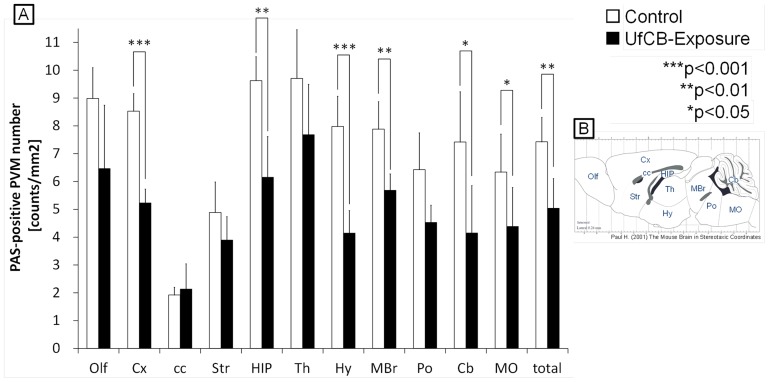
Quantitative observation of PAS-positive PVMs. A: The number of PAS-positive PVMs in each brain region (n = 5/group). Numbers in the following regions were significantly decreased in UfCB-exposed offspring; cerebral cortex (Cx), hippocampus (HIP), hypothalamus (Hy), midbrain (MBr), cerebellum (Cb), and medulla oblongata (MO). Asterisks indicate statistical significance (* P<0.05, **P<0.01, ***P<0.001). Data are shown as mean ±SD. B: The regions of the brain. Abbreviations: Olf, olfactory bulb; Cx, cerebral cortex; cc, corpus callosum; Str, striatum; HIP, hippocampus; Th, thalamus; Hy, hypothalamus; MBr, midbrain; Po, pons; Cb, cerebellum; MO, medulla oblongata.

In the control mice, PAS-positive PVMs around vessels numbered 7.42/mm^2^ for all regions; in contrast with UfCB-exposed mice where PAS-positive PVMs numbered 5.04/mm^2^ (Figure 4, Table S2). The numbers of PAS-positive PVMs were decreased in all regions of the UfCB-exposed offspring except the corpus callosum (cc); the decrease was especially significant in the cerebral cortex (Cx), hippocampus (HIP), hypothalamus (Hy), midbrain (MBr), cerebellum (Cb) and medulla oblongata (MO) (Figure 4).

### Ultrastructural observation by transmission electron microscope (TEM)

Since PAS-positive granules morphologically changed and the number of PAS-positive PVMs was decreased in the UfCB group, we observed the ultrastructure of PVMs by TEM. The cerebral arterioles and venules consisted of a thin endothelium and circularly-arranged smooth muscle cells. On the outside of the vessels, the PVMs were situated between astrocytes and endothelial cells.

**Figure 5:**
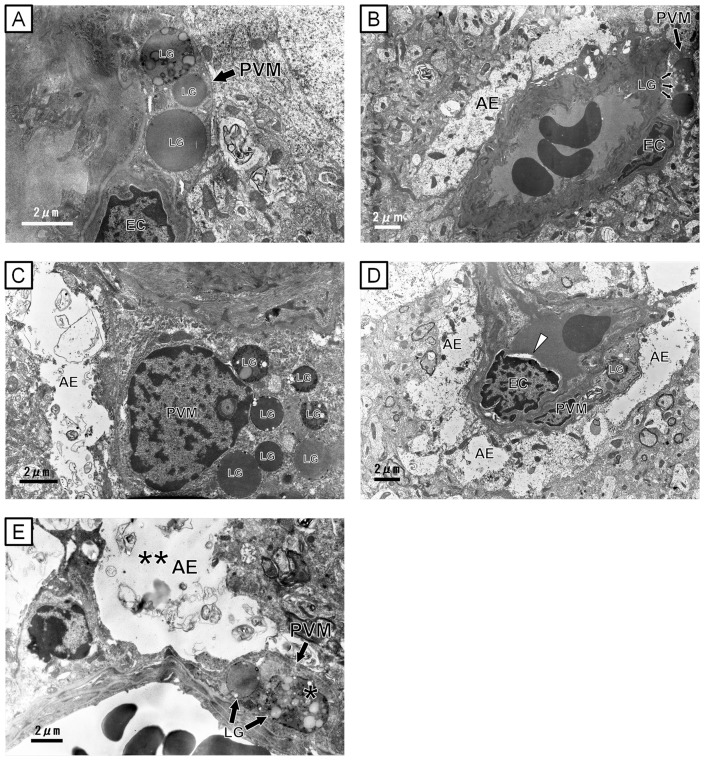
Transmission electron micrographs of perivascular macrophages (PVMs) and astrocytic end-foot. All scale bars represent 2 μm. A–C: Electron micrograph of perivascular regions of the cerebral cortex (grey matter) of (A, B) 6- and (C) 12-week-old mouse in the control group. (A, C) Perivascular macrophages [PVM] were found between endothelial cells and glia limitans with many round lysosomal granules [LG] of moderate intensity and some pale vacuoles. (B, C) The astrocytic end-foot [AE] was attached to a endothelial cells [EC] and a perivascular macrophage. D, E: Electron micrographs of perivascular regions of the cerebral cortex (grey matter) of (D) 6- and (E) 12-week-old UfCB-exposed offspring mouse. (D) Arrow head: Crescent-shaped spaces, an ultrastructural feature of apoptotic bodies [57], of EC were shown. (E) Honeycomb-like structured lysosomal granules (*) were shown in the perivascular macrophages [PVM]. (E) Severe swelling of astrocytic end-feet (**) was found at sites attached to perivascular macrophages with denatured granules.

In control mice, most granules were round, and the contents were homogeneous and high in electron density in both 6- and 12-week-old offspring (Figure 5A, C). There were some inclusion bodies with a vacuolated structure. Only mild swelling of some astrocytic processes and end-feet was observed in the control offspring at both 6- and 12-week-old of age (Figure 5B, C). In UfCB-exposed offspring, granules with honeycomb-like structures were often found in the PVMs in 12-week-old offspring (Figure 5E). Severe swelling of astrocytic end-feet was also found adjacent to the PVMs with denatured granules in 12-week-old offspring (Figure 5E). However, the remarkable change of granules in PVMs and astrocytic end-feet was not observed in the UfCB-exposed offspring 6-week-old offspring. UfCB-like substances were not found in the offspring brains by TEM.

### Relationship between PAS-positive granules and astrocytic GFAP expression

Since degeneration of astrocytic end-feet was observed in the UfCB-exposed offspring (Figure 5), we expected that the phenotype of astrocytes contacting with the vasculature was changed; we therefore analysed the expression of GFAP, which is the most commonly used phenotypic marker of astrocytes subset in the CNS, by immunohistochemistry.

**Figure 6:**
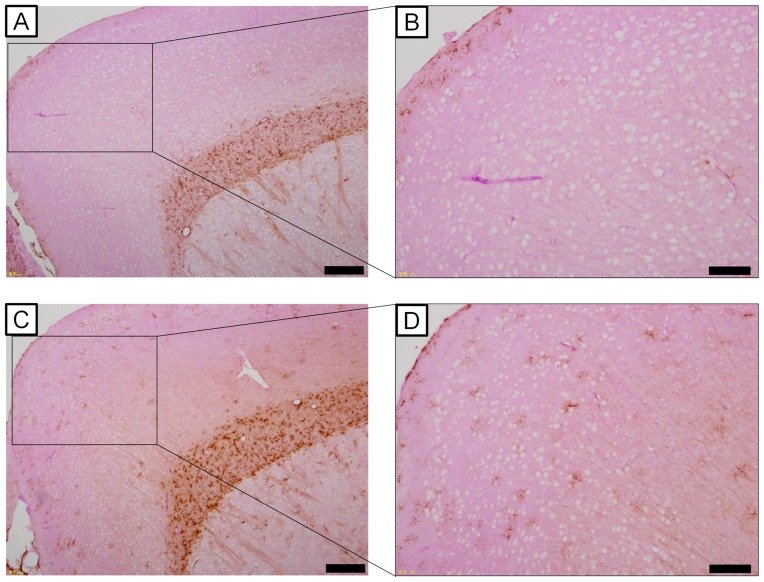
Light micrographs of GFAP-positive astrocytes of the wide-field. Scale bars represent (A, C) 200 m or (B, D) 100 m. A, C: The frontal cortex of 6-week-old male mice of (A) the control group (B) UfCB-exposed offspring. B, D: Enlarged views of A and C. (B) Few GFAP-positive astrocytes were observed in the grey matter in the control group, while (D) many GFAP-positive astrocytes were detected in the grey matter in UfCB-exposed offspring. GFAP-positive astrocytes were not observed at any sites attached to blood vessels with PVMs with small (approximately 1 m) PAS-positive granules (Figure 7A), but were found at blood vessels with PVMs that had enlarged (approximately 2-3 m) PAS-positive granules in the UfCB-exposed offspring (Figure 7B, C). Moreover, GFAP-positive astrocytic end-feet were detected at sites attached to PVMs with enlarged PAS-positive granules (Figure 7E, G, H), and not surrounding PVMs with small PAS-positive granules (Figure 7F, I) around one blood vessel in the UfCB-exposed offspring (Figure 7D-I). These results suggested that the increase in the expression level of GFAP in astrocytic end-feet around blood vessels in the grey matter was correlated with enlargement of PAS-positive granules of PVM .

In both groups, GFAP-positive astrocytes were focally prominent in the white matter (Figure 6A, C). In UfCB-exposed offspring, however, GFAP-positive astrocytes were clearly detected in the perivascular region of the grey matter (Figure 6C, D). In addition, astrocytic GFAP-positive processes completely surrounded neuronal bodies in the frontal cortex of the grey matter (Figure 6C, D).

**Figure 7:**
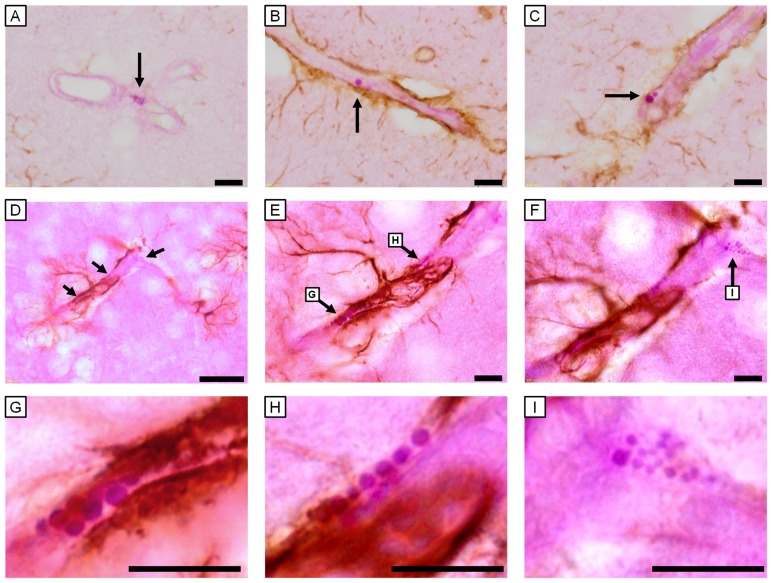
Light micrographs of GFAP-positive astrocytes and PAS-positive PVMs of 6-week-old male mice. All scale bars represent 10 μm. (A–C) 6-μm paraffin sections and (D–I) 10-μm frozen sections. A: PVM surrounding the cerebral blood vessels of a 6-week-old control mouse. GFAP-positive astrocytes were very few in number at sites attached to blood vessels with PVMs with small (approximately 1 μm) PAS-positive granules (arrow). B, C: PVM surrounding cerebral blood vessels of a mouse in the UfCB group. The PVM possessed enlarged granules (arrows). Many GFAP-positive astrocytes were observed at sites attached to PVMs with enlarged granules. D: GFAP-positive astrocytes and PAS-positive PVM (arrows) surrounding cerebral blood vessels of mouse in the UfCB group. E–I: Enlarged views of (D). Around one cerebral vessel, GFAP-positive astrocytic end-feet were detected (E, G, H) at a site attached to PVM with enlarged PAS-positive granules (G, 2.2 μm; H, 1.6 μm), but (F, I) not surrounding PVMs with small PAS-positive granules (smaller than 1.1 μm).

GFAP-positive astrocytes were not observed at any sites attached to blood vessels with PVMs with small (approximately 1 μm) PAS-positive granules (Figure 7A), but were found at blood vessels with PVMs that had enlarged (approximately 2–3 μm) PAS-positive granules in the UfCB-exposed offspring (Figure 7B, C). Moreover, GFAP-positive astrocytic end-feet were detected at sites attached to PVMs with enlarged PAS-positive granules (Figure 7E, G, H), and not surrounding PVMs with small PAS-positive granules (Figure 7F, I) around one blood vessel in the UfCB-exposed offspring (Figure 7D–I). These results suggested that the increase in the expression level of GFAP in astrocytic end-feet around blood vessels in the grey matter was correlated with enlargement of PAS-positive granules of PVM.

## Discussion

In the present study, we examined the effect of maternal exposure to UfCB on PVMs and surrounding tissue in murine CNS using PAS staining, transmission electron microscopy and GFAP immunohistochemistry. First, we counted PAS-positive PVMs quantitatively in each brain region with a simple method for staining large quantities of tissue sections uniformly and entirely. PAS staining can selectively detect PVMs and meningeal macrophages in the CNS. Only the PAS-positive cells located with blood vessels in the brain parenchyma were counted in order to quantitatively analyse the PVMs in the CNS. Although we can detect murine and human PVMs with scavenger receptors (CD204, CD163) [2], [15], these molecules are not so selective because it is also expressed on the microglia in the CNS, especially under inflammatory conditions [15], [40]. Previous studies have suggested that PVMs were predominantly present in the grey matter and that the corpus callosum, one of the typical regions in the white matter, had a smaller number of PVMs [1]. The number of PVMs was positively correlated with capillary density in each region. Our quantitative data showed that the frequency of PAS-positive PVM appearance was 4-fold higher in the cerebral cortex than in the corpus callosum; likewise, capillary density was also reported to be about 4-fold higher in the cerebral cortex than in the corpus callosum [41]. PVMs are positive for PAS in physiological and mild-pathologic conditions such as Tay-Sachs disease [42], but may be negative for PAS when they are severely degenerated in conditions such as Sandhoff's disease [42]. On the other hand, the number of PVMs are greatly increased and accumulated around blood vessels in experimental allergic encephalomyelitis [8].

Our data showed that PVM granules were enlarged and that the number of PAS-positive PVMs indicated a decrease in “normal” PVMs in a wide area of the CNS after maternal UfCB exposure. Generally, PVM granules are enlarged in mild pathological and aging conditions [42], [43], [44], presumably by the uptake and accumulation of substances from plasma [43]. More severely denatured PVMs cannot retain spherical (lipid and waste products) granules, and PAS-positive granules have been shown to become negative for PAS-staining [42], [45]. These reports suggest that a decrease in PAS-positive PVMs is generally well-correlated with dysfunction of the cells. Furthermore, our electron microscopy results, where some honeycomb-like structures were observed in the granule, support the denaturation of PVM granules after growth (12-week-old offspring) in the UfCB group.

An increase in GFAP expression in the cells surrounding blood vessels in the cerebral cortex suggested that the blood vessels may be damaged in UfCB-exposed offspring. GFAP is an intermediate filament protein, which is the most common phenotypic marker labelling of astrocytes under conditions of denaturation or inflammation [46], [47]. Astrocytes highly express GFAP in their end-feet and extend it to injured regions, where cerebral blood vessels or neuronal cells are injured in pathological conditions such as infection and transient ischemia [46], [48], [49]. In addition, the swelling of astrocytic end-feet in UfCB-exposed offspring, as observed by electron microscopy, was similar to a feature of ischemia-related blood vessel damage [46], [47]. These data also suggest that maternal UfCB exposure induces persisting alteration of the phenotype of PVMs and attached astrocytes in the brains of mouse offspring.

Interestingly, the increase in astrocytic GFAP expression level was closely related to the enlargement of granules in the attached PVMs. A decrease in the number of normal PVMs suggests PVM dysfunction in the UfCB group, the cause of which may be the invasion of foreign matter or pathogens to the brain parenchyma [3], [43], accumulation of waste products [3], a decrease in immunocompetence in the surrounding blood vessels [3], [13], and/or damage to blood vessels and weakening of the BBB function [15]. To protect against and restrict the spread of infectious agents and inflammatory cells in the CNS parenchyma, an astrocyte extends its end-foot to the vessel when there is an increase in the expression level of astrocytic GFAP [46], [49]. PVMs with enlarged granules may be associated with the attraction of astrocytic end-feet toward themselves in UfCB-exposed offspring. GFAP-positive astrocytic end-feet were observed around PVMs with enlarged granules.

The present study also contributed to elucidating the mechanism underlying the effect of maternal exposure to ultrafine particles in the atmospheric environment. It is well-known that the core of combustion-derived particles is composed of UfCB [50], which represents relevant surrogate model particles for airborne fine (PM_2.5_) and ultrafine particles [51]. Further investigation is needed to clarify which direct or indirect effect of UfCB is the main contributor to the effect on PVMs and surrounding astrocytes in mouse offspring. Ultrafine particles with a diameter of <200 nm may transfer from the pregnant body to offspring by passing through the placenta [29], [52] and may directly affect the development of offspring. Alteration in the pregnant body, such as an increase in circulating cytokines or other secondary messengers that are activated in response to inflammation and/or oxidative stress, may also influence the development [53], [54], [55]. Our results demonstrate the need to find a means of preventing and controlling the developmental effect of maternal exposure to ultrafine particles on the CNS, especially in the maintenance of brain perivascular regions. The previous study reported that the changes of gene expression in olfactory bulb, where is one of the brain region, by exposure to diesel exhaust were prevented by enrichment rearing environment [56]. The living environment during perinatal period is of interest for preventing the development effects of ultrafineparticles.

In summary, the present study showed that exposure of pregnant mothers to UfCB degenerated PVM granules and decreased the number of normal PVMs of offspring. An increase in the expression level of GFAP was also shown in the astrocytes surrounding PVMs with enlarged granules in UfCB-exposed offspring. The phenotypic changes in PVMs and astrocytes indicate that maternal UfCB exposure may alter brain blood vessels and be associated with the risk of brain dysfunction and disorders in future offspring. It would be necessary to clarify the mechanisms underlying the effect of UfCB on the astrocytes and CNS function of offspring.

## Supporting Information

Table S1 **Total number of PAS-positive PVMs in each sample.**
(DOC)

Table S2 **Number of PAS-positive PVMs in each brain region.** Data are presented as mean ± SD. Abbreviations: Olf, olfactory bulb; Cx, cerebral cortex; cc, corpus callosum; Str, striatum; HIP, hippocampus; Th, thalamus; Hy, hypothalamus; MBr, midbrain; Po, pons; Cb, cerebellum; MO, medulla oblongata.
(DOC)
