# Supplementary material for: Effects of Maternal Exposure to Ultrafine Carbon Black on Brain Perivascular Macrophages and Surrounding Astrocytes in Offspring Mice
Source: PLoS One. 2014 Apr 10;9(4):e94336. doi: 10.1371/journal.pone.0094336 (PMC3983141; doi:10.1371/journal.pone.0094336)
Supplement: Table S1 — Total number of PAS-positive PVMs in each sample. (DOC) [file pone.0094336.s001.doc]

**Supplementary Table S1.**

|  | **Control** | | | | | |
| --- | --- | --- | --- | --- | --- | --- |
| **Sample ID** | **C1** | **C2** | **C3** | **C4** | **C5** | **Sum** |
| **Total counts** | 991 | 890 | 1225 | 1036 | 1206 | 5348 |
| **Total area [mm^2^]** | 156.6 | 132.0 | 144.1 | 132.6 | 155.7 | 721.0 |

|  | **UfCB-Exposure** | | | | | |
| --- | --- | --- | --- | --- | --- | --- |
| **Sample ID** | **E1** | **E2** | **E3** | **E4** | **E5** | **Sum** |
| **Total counts** | 677 | 770 | 690 | 943 | 998 | 4078 |
| **Total area [mm^2^]** | 157.7 | 163.4 | 154.4 | 164.4 | 166.3 | 806.2 |
