# Supplementary material for: Effects of Maternal Exposure to Ultrafine Carbon Black on Brain Perivascular Macrophages and Surrounding Astrocytes in Offspring Mice
Source: PLoS One. 2014 Apr 10;9(4):e94336. doi: 10.1371/journal.pone.0094336 (PMC3983141; doi:10.1371/journal.pone.0094336)
Supplement: Table S2 — Number of PAS-positive PVMs in each brain region. Data are presented as mean ± SD. Abbreviations: Olf, olfactory bulb; Cx, cerebral cortex; cc, corpus callosum; Str, striatum; HIP, hippocampus; Th, thalamus; Hy, hypothalamus; MBr, midbrain; Po, pons; Cb, cerebellum; MO, medulla oblongata. (DOC) [file pone.0094336.s002.doc]

**Supplementary Table S2.**

|  | **PAS-positive PVMs [count/mm^2^]** | | | | | |
| --- | --- | --- | --- | --- | --- | --- |
|  | **Olf** | **Cx** | **cc** | **Str** | **HIP** | **Th** |
| **Control** | 8.89 ± 1.10 | 8.53 ± 0.63 | 1.92 ± 0.27 | 4.89 ± 1.10 | 9.62 ± 0.86 | 9.70 ± 1.75 |
| **UfCB** | 6.47 ± 2.27 | 5.23 ± 0.49 | 2.14 ± 0.90 | 3.90 ± 0.84 | 6.15 ± 1.46 | 7.69 ± 1.80 |

|  | **PAS-positive PVMs [count/mm^2^]** | | | | | |
| --- | --- | --- | --- | --- | --- | --- |
|  | **Hy** | **MBr** | **Po** | **Cb** | **MO** | **Total** |
| **Control** | 7.98 ± 1.09 | 7.88 ± 0.99 | 6.42 ± 1.32 | 7.42 ± 1.81 | 6.34 ± 1.37 | 7.42 ± 0.88 |
| **UfCB** | 4.15 ± 0.81 | 5.69 ± 0.26 | 4.53 ± 1.70 | 4.15 ± 1.40 | 4.39 ± 1.07 | 5.04 ± 0.78 |
